# ^131^I thyroid activity and committed dose assessment among family members of patients treated with radioactive iodine

**DOI:** 10.1007/s00411-020-00860-z

**Published:** 2020-06-20

**Authors:** K. Brudecki, A. Kluczewska-Gałka, P. Zagrodzki, B. Jarząb, K. Gorzkiewicz, T. Mróz

**Affiliations:** 1 Polish Academy of Sciences, Institute of Nuclear Physics, Radzikowskiego 152, 31-342 Kraków, Poland; 2 Department of Nuclear Medicine and Endocrine Oncology Comprehensive Cancer Center, M. Skłodowska-Curie Memorial Institute of Oncology, Wybrzeże Armii Krajowej 15, 44-101 Gliwice, Poland; 3 Department of Food Chemistry and Nutrition, Medical College Jagiellonian University, Medyczna 9, 30-688 Kraków, Poland; 4 Institute of Physics, Jagiellonian University, Łojasiewicza 11, 30-348 Kraków, Poland

**Keywords:** ^131^I, Family members, Whole body counting, Dosimetry

## Abstract

The main goal of the present study was estimation of an internal contamination of ^131^I among family members of patients treated with radioactive iodine. Thyroid activity measurements of ^131^I in examined volunteers were performed using a whole-body spectrometer at the institute of nuclear physics, Polish academy of sciences. During this research, 20 relatives of patients treated with ^131^I were examined: eight women and 12 men with an age in the range from 3 to 72 years. In the case of nine individuals, the activity of ^131^I in the thyroid was below the detection limit, but among the remaining 11 individuals, the activity varied from (9 ± 3) Bq up to (1140 ± 295) Bq. Subsequently, based on the measurements of thyroid ^131^I activities, the corresponding doses were assessed. The highest estimated effective dose reached 218 μSv, while the thyroid equivalent dose was 2.4 mSv. In addition, the experimental data obtained were statistically analysed together with the results of surveys of the individuals participating in the study by means of correspondence analysis and nonparametric tests: Mann–Whitney, gamma, *χ*^2^ and Yule Phi coefficient. These analyses revealed relationships between ^131^I activities in the thyroids of the examined individuals and their housing conditions as well as consumption of meals prepared by the patients.

## Introduction

Since the very beginning of nuclear medicine (the 30–40 s of the 20th century), ^131^I has been playing a key role. ^131^I was discovered by Glenn Seaborg and John Livingood in 1938 at the University of California, Berkeley. The first reports about its application in medicine appeared less than eight years later, when Hertz and Roberts, and Seidlin and co-workers successfully applied ^131^I in treatment procedures of Graves’ disease and thyroid cancer (Hertz and Roberts 1946; Seidlin et al. 1946). Currently, ^131^I is used in thyroid and whole-body scintigraphy, and in treatment of hyperthyroidism and thyroid cancer. In the case of thyroid disease, in Poland, single activities of ^131^I administered to patients are in the range from 0.05 mCi (1.85 MBq) in the case of thyroid scintigraphy, up to 250 mCi (9.25 GBq) during cancer treatment.

The above-mentioned medical interventions like scintigraphy and hyperthyroidism treatment allow performing limited ambulatory care, which means that after the application of ^131^I, patients may return to their homes. In the case of thyroid cancer treatment, it is necessary to isolate patients in the hospital for a few days after ^131^I application (usually 3–5 days). In Polish hospitals, during this period, constant monitoring of the dose rate from the patient’s body is performed. When the dose rate drops below 5 µSv h^−1^ at a distance of 2 m, the patient may qualify for discharge from hospital. Such a dose rate corresponds to an activity of about 400 MBq accumulated in the patient’s body. This activity level (400–600 MBq) is used as the patient discharge limit in most European Union member states (EURATOM 1997; ICRP 2004). Another criterion is the dose rate of radiation emitted from the patient’s body. In many European countries (e.g., Germany, and in contrast to Poland) the dose rate limit is set at 3.5 µSv h^−1^ (measured at a distance of 2 m from the patient) and the hospitalization period is set to at least 2 days. Different regulations are used in Japan, where the patient can be released from hospital if the ^131^I activity in his body is less than 500 MBq or the dose rate measured at a distance of 1 m is less than 30 µSv h^−1^. In the United States, patients’ release criteria are defined by the US Nuclear Regulatory Commission (USNRC 1997a, b). In general, release criteria are based on physical half-life, activity or dose rate. Based on these criteria, the International Commission on Radiological Protection recommends patients to be released from hospital if their residual activity of ^131^I is less than 800 MBq (ICRP 2004). In addition, patients should receive basic instructions regarding radiological protection, which should be followed after returning home. In this context, the most important instructions for patients are isolation from other members of the family for about 2 weeks, lack of contact with children and pregnant women, utilization of their own toilet and kitchen utensils as well as special care for personal hygiene and caution when urinating.

However, there is a potential risk that patients may neglect or even totally desist obeying these radiological protection rules. So far, the approach to radiation protection of family members’ patients treated with radioactive iodine was based only on the assessment of external doses (Mathieu et al. 1999; Pant et al. 2005; Kocovska et al. 2011a, b; Zehtabian et al. 2017). Nonetheless, ^131^I is being excreted from the patient’s body (mainly in sweat and urine) and easily sublimes, which may cause a potential risk that it is inhaled relatives and accumulated in their thyroids. Consequently, the main goal of the present study was an estimation of internal contamination with ^131^I among family members of patients treated with radioactive iodine and assessment of corresponding radiation doses.

## Materials and methods

### Volunteers

In this study, 20 relatives of patients treated with ^131^I were examined: eight women and 12 men at an age in the range from 3 to 72 years. These individuals were family members of patients treated for hyperthyroidism (six cases) or thyroid cancer (14 cases). Predominantly, they were patients’ partners or children (four children under 18 years of age). The study was approved by the Bioethics Committee at the Regional Chambers of Physicians and Dentists in Krakow, Poland (decision number 111/KBL/OIL/2015, dated 09.09.2015).

### ^131^I activity measurements

Thyroid activity measurements of ^131^I in examined volunteers were performed using the whole-body spectrometer at the Institute of Nuclear Physics, Polish Academy of Sciences. The shielding of the spectrometer was purposely designed allowing to place and measure the whole body of an adult person: the shielding is 2 m long, 1.2 m wide and 1.3 m high. The shield is made of steel from the 19th century, free from ^60^Co traces to keep radiation background low, and the thickness of its walls is about 17 cm, while its mass is about 18 tons (Mietelski et al. 2013).

The activity of ^131^I was measured using a low-background gamma-ray spectrometer equipped with an n-type germanium detector (HPGe, GMX-30190-P by Ortec with 30% relative efficiency). Data acquisition and analysis were performed using Canberra equipment and software (Multiport II, Genie-2000). Quantification of ^131^I activity in thyroids was carried out by measuring the 364.49 keV gamma-line.

Calibration of the detector was performed with an adult human thyroid phantom containing an ^131^I solution with an activity of 6 kBq ± 0.6%. The dimensions of the phantom are 5.5 cm long, 2.5 cm wide and 2 cm high. The average detector efficiency was estimated to be (0.31 ± 0.08) %. The estimated uncertainty is relatively high, namely 25%, because it takes into account the large uncertainty due to the relative position of the thyroid gland and the detector (Kierepko et al. 2014). This high uncertainty of the efficiency propagates to a relatively high uncertainty of ^131^I activity in the thyroid gland.

A single measurement lasted about 60 min, and the detector was placed from 2 to 4 cm from thesurface of the neck. Typically, in the measurements a limit of detection (LOD) of about 4–6 Bq was achieved, depending on the measurement parameters described above. Measurements of ^131^I activity in relatives’ thyroids were performed 7 days after discharge of patients from hospital.

### Questionnaire surveys

Every person who took part in the study was asked to complete a questionnaire that consisted of four main parts. The first part included general questions about height, weight and gender, while the second part described frequency and character of interactions with the treated person. The third part focused on housing conditions like living in the city or on the countryside, living in a single-family house or in a flat, surface area of apartment, number of rooms or bathroom sharing with the patient. In the last part the participants were asked about their food habits (e.g., whether they consumed meals prepared by patient). Apart from anthropometric parameters, all other data from the questionnaire were strictly categorized by assigning them either logical values: yes = 1 or no = 0 (for dichotomous variables), or more numerically ordered values (for multilevel variables), thus bringing about dichotomous and multilevel categorical parameters, respectively.

### Statistical approach

To quantify any dependencies between the investigated parameters, 20 complete survey forms were studied with a statistical correspondence analysis (CA) method. The nominal or ordinal data from the questionnaire (the dichotomous and multilevel categorical parameters mentioned above) were gathered in a contingency table together with the thyroid activities measured in the individuals. The results on ^131^I activity were then transformed into an ordinal scale (four categories) according to the following algorithm: (1) low activities (< 11 Bq), (2) moderate activities (≥ 11 Bq and < 150 Bq), (3) elevated activities (≥ 150 Bq and < 600 Bq), (4) highly elevated values (> 1100 Bq). All these parameters formed a multidimensional space of original scores. The analyses of coordinates of the dichotomous and multilevel categorical parameters in the coordinate system of the CA model, generated in the reduced (two-dimensional) space determined by the first two new dimensions of this model, allowed to reveal the structure of associations between parameters. In this work, CA was constructed under the condition that its first two dimensions should explain at least 50% of the total inertia in the original set of parameters. Thus, the parameters or their categories with the lowest quality of representation were subsequently discarded as well as associations in the CA model depending only on single coincidence. Those parameters with large absolute values of their coordinates (> 0.3) in the CA model were assumed to be associated with one another. To express the strength of bivariate associations, for the pairs of associated parameters, the algebraic products of their corresponding coordinates and cosine of the corresponding angle were calculated (these coefficients are called the association weights). The “corresponding angle” was defined as the angle determined by two lines connecting the origin with coordinates of both parameters on the CA coordinates plot. Apart from CA, the gamma correlations (*R*_γ_) were computed for the pairs of categorical multilevel variables, while for dichotomous variables the *χ*^2^ test and Yule Phi coefficient were calculated. For thyroid activities, categories (2), (3) and (4) were combined, thus, this variable became a dichotomous one. The difference of thyroid activities (categorized into four groups) between family members of patients either with cancer or with hyperthyroidism was checked using the Mann–Whitney *U* test. To estimate the mean values of thyroid activities in the above-mentioned two groups of family members, data were transformed in logarithms and retransformed after calculation, because the parameter had a non-Gaussian distribution. Therefore, the data are shown as mean and confidence interval. In the calculations, the data below the LOD were substituted with the value of LOD × 2^−0.5^.

The statistical analyses were carried out using the STATISTICA v. 12 package (Statsoft, Tulsa, OK, USA) and the software delivered by MP System Co. (Chrzanów, Poland). The latter was used to calculate correlation weights for the pairs of parameters in the CA model.

### Doses estimation

In the case of individuals with detected ^131^I in the thyroid gland, complete uptake of ^131^I by the respiratory system as well as its activities in other organs were calculated. Calculations were based on the iodine biokinetics model developed by Leggett (Leggett 2010, 2017) in combination with the Human Respiratory Tract Model (ICRP 1994, 2002) and the Gastrointestinal Tract Model (ICRP 1979) developed by the International Commission on Radiological Protection (ICRP). For computer modeling, the SAAM II software from Epsilon Group was used (Barrett et al. 1998). The method used to calculate radiation doses was presented in detail in previous papers (Li 2018; Brudecki et al. 2014, 2017a, b, 2018a, b, 2019).

## Results and discussion

In the present study, 20 individuals, i.e., relatives of patients treated with ^131^I, were examined. Results above the detection limit were obtained in 11 cases (55%), and they were in the range (9 ± 3) Bq up to (1140 ± 295) Bq. The exact results are presented in Table 1. These individuals were family members of patients treated for hyperthyroidism (six cases) or thyroid cancer (14 cases). Among the first group, results above detection limit were achieved for four individuals (67%), and the mean 131I activity for them was 41.7 Bq with a 95% confidence interval of (4.6; 376.0) Bq. Values above the detection limit in the second group were observed in seven cases (50%) with an average value 14.7 Bq and a 95% confidence interval of (2.3; 94.5) Bq.

**Table 1:** ^131^I thyroid activity among family members of patients treated with radioactive iodine (thyroid activity uncertainties were calculated by means of uncertainty propagation method)

Code	Gender	Personal relationship with patients treated with ^131^I	Type of treatment	^131^I thyroid activity [Bq]
A1	M	Husband	Thyroid cancer	9 ± 3
A2	M	Son	Thyroid cancer	< 4
A3	F	Daughter	Hyperthyroidism	51 ± 14
A4	M	Husband	Thyroid cancer	< 5
A5	F	Wife	Thyroid cancer	341 ± 88
A6	M	Husband	Hyperthyroidism	1140 ± 295
A7	F	Daughter	Thyroid cancer	20 ± 6
A8	M	Husband	Hyperthyroidism	98 ± 25
A9	M	Husband	Thyroid cancer	151 ± 39
A10	F	Wife	Thyroid cancer	10 ± 3
A11	F	Sister	Thyroid cancer	< 4
A12	M	Husband	Thyroid cancer	< 4
A13	F	Wife	Thyroid cancer	557 ± 144
A14	M	Husband	Hyperthyroidism	72 ± 19
A15	M	Son	Hyperthyroidism	< 5
A16	M	Husband	Hyperthyroidism	< 6
U1	F	Daughter	Thyroid cancer	58 ± 15
U2	M	Son	Thyroid cancer	< 10
U3	F	Daughter	Thyroid cancer	< 11
U4	M	Son	Thyroid cancer	< 5

*A*, adults, *U*, underage, *M*, male, *F*, female

As can be also inferred from Table 1, four children and 16 adults took part in this study. In the group of adults, an activity of ^131^I above detection limit was found in ten (62.5%) of the examined individuals. In these individuals, measured activities ranged from (9 ± 3) Bq up to (1140 ± 295) Bq. In the group of children, in one case (25%) the activity of ^131^I in the thyroid was above the detection limit and reached (58 ± 15) Bq. The difference in the number of results above detection limit—25% vs. 62.5%—as well as the difference in maximum activities (51 ± 14 Bq vs. 1140 ± 295 Bq) observed among children and adults might be explained by an increased concern about the safety of children in all aspects of everyday life.

Interestingly, the maximum measured activity reached, 1140 ± 295 Bq, is relatively high. For comparison, in a separate study it was shown that the maximum activity of ^131^I in the thyroid of medical staff who work with radioiodine on a daily basis reached only 217–457 Bq (Brudecki et al. 2017a, 2018b). The reason for such a high ^131^I activity in the thyroid of the husband of a female patient (see Table 1) may be the fact that the treated patient was bedridden and in need of continuous care by the tested relative.

As mentioned earlier, the measured ^131^I activities were statistically analysed along with the results of surveys of the individuals participating in the study, by means of CA and nonparametric tests: Mann–Whitney, gamma, *χ*^2^ and Yule Phi coefficient. The results are summarized in Tables 2, 3

**Table 2:** Results of statistical analyses for dichotomous variables

Pairs of associated parameters		*χ*^2^ value	Significance level (*p*)	Yule phi coefficient
Thyroid activity	Living in city or in countryside	5.05	0.025	0.503
Thyroid activity	Surface area^a^ of apartment	5.09	0.024	− 0.504
Thyroid activity	Consumption of meals prepared by patient	7.01	0.008	0.592
Thyroid activity	living in single-family house or in flat	8.81	0.030	0.664
Thyroid activity	Bathroom sharing with the patient	13.39	0.000	0.818

^a^categorized sum of surfaces of all floors

**Table 3:** Association weights based on the correspondence analysis model [Thyroid activity III—elevated activities (≥ 150 and < 600 Bq), Thyroid activity IV—highly elevated values (> 1100 Bq)]

Pairs of associated parameters		weight
Thyroid activity IV	Consumption of meals prepared by patient	0.96
Thyroid activity III	Number of rooms in apartment	0.87
Thyroid activity IV	Bathroom sharing with the patient	0.87
Thyroid activity IV	Frequent contact with patients	0.85

Based on the performed analysis it can be concluded that the level of ^131^I activity in the thyroid of the examined individuals is primarily affected by their housing conditions. The gamma correlation analysis allowed to quantify the relationship between thyroid activity and the number of rooms in the apartment (*R*_γ_ = – 0.767, *p* < 0.05). Next, *χ*^2^ and Yule Phi tests suggested concomitance between ^131^I activity in the thyroid and the following parameters: living in a city or on the countryside, the surface area of the apartment, consumption of meals prepared by the patient, living in a single-family house or in a flat, as well as sharing a bathroom with the patient (Table 2). This means that the ^131^I activity depends on whether the patients and their relatives live in a city or on the countryside. Specifically, it was found that ^131^I activity in thyroids of relatives living in the city was higher than that of relatives living on the countryside. This could be explained taking into consideration that people in cities have typically less living space available than people on the countryside (for example in terms of surface area of the rooms, number of bathrooms). Moreover, higher ^131^I activities were observed in relatives living in flats (as compared to those living in single family houses), as well as in the situations when the meals were prepared by patients and the bathroom was shared with them. Conducted CA analyses confirmed the above-mentioned findings, and in addition, revealed a connection between ^131^I activity in relatives’ thyroid and the frequency of their contacts with the patients (Table 3).

The difference in thyroid activities of family members of patients either with cancer or hyperthyroidism was not statistically significant. However, the number of examined individuals was low and, consequently, one should treat this result with caution.

The highest observed activity in the thyroid (1140 ± 295 Bq) corresponds to an effective dose of only 218 µSv, while the thyroid equivalent dose was 2.4 mSv. In the group of children, the maximum effective dose and thyroid equivalent dose reached 13 µSv and 0.16 mSv, respectively. These doses are relatively low, keeping in mind that the maximum effective dose of 218 µSv is only about 9% of the mean annual effective dose due to natural radiation sources in Poland, which may suggest that there will be no discernible negative consequences for the health of the examined individuals. In addition, the estimated doses are also significantly lower than the external doses found in other studies for relatives of patients treated with ^131^I (Mathieu et al. 1999; Pant et al. 2005; Kocovska et al. 2011a, b; Zehtabian et al. 2017).

## Conclusions

So far, the issue of internal contamination was mainly associated with people who work with open radioactive sources on a daily basis (e.g., medical staff). The current study showed that internal contamination may also affect relatives of patients treated with radiopharmaceuticals. Even though ^131^I activities in thyroids of the investigated relatives could be detected in some cases (up to about 1000 Bq), the related doses are rather low, i.e., less than 218 µSv, which is less than about 9% of the mean annual effective dose due to natural radiation sources in Poland, which is about 2.48 mSv per year (Janik and Tokonami 2009). In the light of current knowledge it is concluded that internal doses received by family members of patients treated with ^131^I will not result in any discernable negative health consequences.

In addition, the results of the statistical analyses performed in the present study revealed relationships between ^131^I activities in the thyroids of some of the investigated individuals and their housing conditions (number of rooms, surface area of apartments, whether or not a bathroom is shared with a patient discharged from hospital after treatment with ^131^I) as well as consumption of meals prepared by the patient. Knowledge of such facts may lead to an improvement in the radiological protection of patients’ relatives in the future.
